# Palliative Care Approach in a Case of Severe Nonketotic Hyperglycinemia With Long Survival: A Case Report

**DOI:** 10.7759/cureus.91636

**Published:** 2025-09-04

**Authors:** Diana Pereira, Tiago Santos, Patrícia Janeiro, Maria João Palaré

**Affiliations:** 1 Pediatrics Department, Hospital Santa Maria, Unidade Local de Saúde Santa Maria, Lisbon, PRT; 2 Pediatric Neurology Unit, Pediatrics Department, Hospital Santa Maria, Unidade Local de Saúde Santa Maria, Lisbon, PRT; 3 Hereditary Metabolic Diseases Unit, Pediatrics Department, Hospital Santa Maria, Unidade Local de Saúde Santa Maria, Lisbon, PRT; 4 Pediatric Palliative Care Support, Pediatrics Department, Hospital Santa Maria, Unidade Local de Saúde Santa Maria, Lisbon, PRT

**Keywords:** epilepsy, multidisciplinary approach, neurodevelopmental delay, nonketotic hyperglycinemia, palliative care

## Abstract

Nonketotic hyperglycinemia (NKH) is a rare autosomal recessive metabolic disorder characterized by defective glycine cleavage, resulting in glycine accumulation, particularly in the central nervous system. The severe neonatal form typically presents with early-onset encephalopathy, refractory epilepsy, and poor neurodevelopment, often leading to early mortality. We report a male patient with genetically confirmed severe NKH who is currently alive at seven years of age - one of the longest reported survivals for this form. He presented within the first day of life with hypotonia, myoclonic seizures, and coma. Diagnosis was supported by markedly elevated glycine levels in plasma, urine, and cerebrospinal fluid (CSF), with a CSF/plasma glycine ratio of 0.19, as well as characteristic MRI findings. Despite early institution of glycine-lowering agents and antiepileptic therapy, he developed refractory epilepsy and profound neurodevelopmental delay. Comprehensive and continuous multidisciplinary care, including pediatric palliative care, was established early and coordinated across care settings. Although there is no curative therapy for severe NKH, this case illustrates that early and sustained multidisciplinary palliative care can significantly enhance the quality of life and potentially extend survival in affected patients. While developmental outcomes remained poor, the integrated care model minimized hospitalizations, stabilized clinical status, and provided essential family support.

## Introduction

Nonketotic hyperglycinemia (NKH) is a rare autosomal recessive disorder caused by defects in the glycine cleavage system. The resulting accumulation of glycine in the central nervous system disrupts glycinergic neurotransmission [1]. NKH is classified into severe and attenuated forms. The severe form is characterized by early-onset symptoms, refractory epilepsy, and absent neurodevelopmental progress. The attenuated form presents more variably, with later presentation, some developmental acquisition, and milder epilepsy [2].

Approximately 85% of the children with severe disease present in the first days of life with poor feeding, lethargy and hypotonia, rapidly progressing to coma, myoclonic seizures, and hiccups. Respiratory depression occurs in the first two weeks of life, with subsequent recovery. Mortality during this period is high despite supportive therapy. Those who survive develop severe intellectual disability and intractable epilepsy. Some patients have a hypoplastic corpus callosum, and most patients have restricted diffusion on MRI in long myelinated tracts. Laboratory findings consist of moderate to severe hyperglycinemia and hyperglycinuria, elevated cerebrospinal fluid (CSF) glycine levels, and an elevated CSF/plasma glycine concentration ratio, with a CSF/plasma glycine ratio > 0.08 being indicative of NKH, and values ≥ 0.15 consistent with severe forms [1,3].

The management of NKH is supportive. Glycine-lowering agents and glycine dietary restriction ameliorate symptoms but do not affect disease progression [1-3]. Given the poor prognosis, early multidisciplinary pediatric palliative care is essential.

## Case presentation

**Figure 1:**
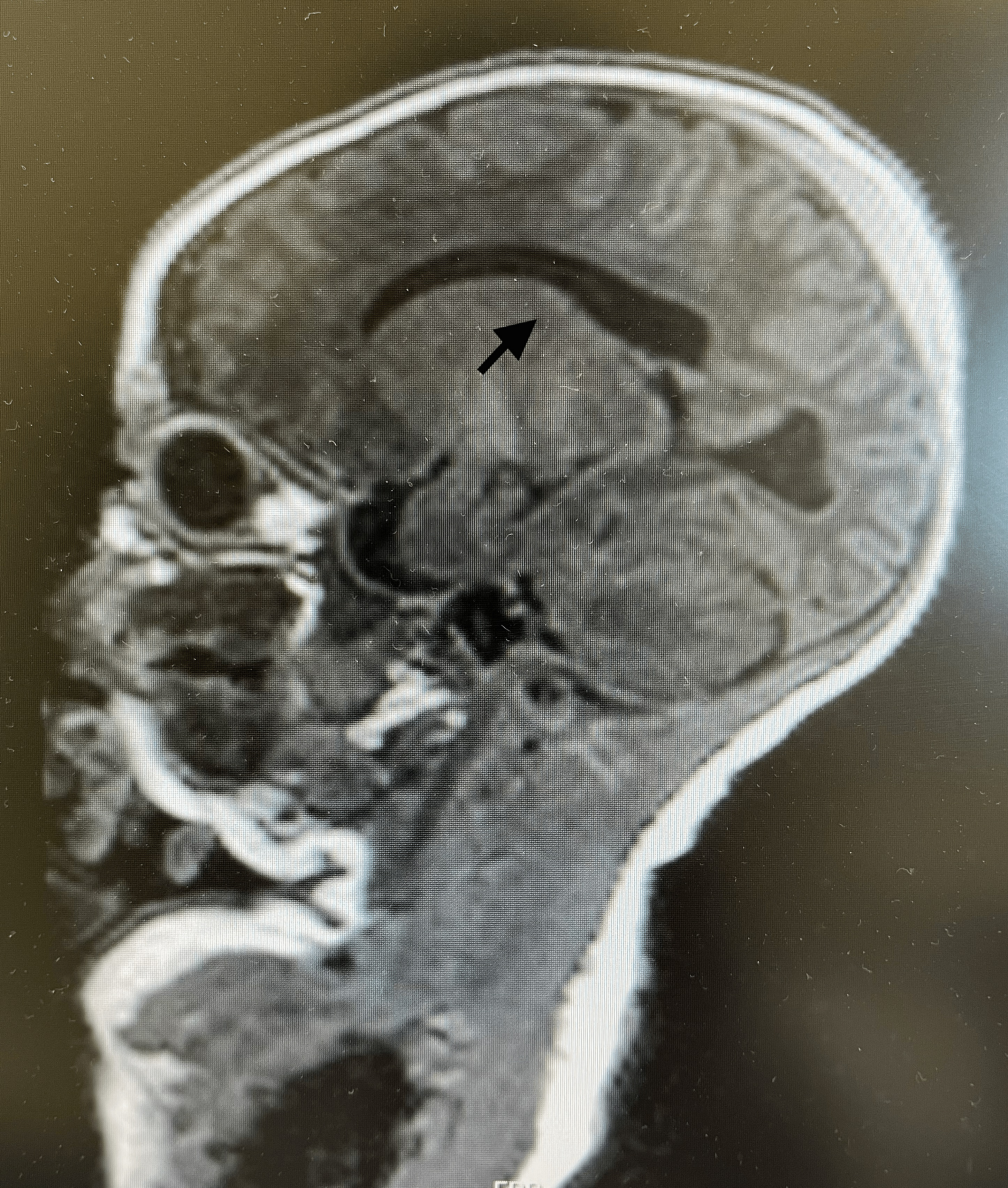
Brain MRI showing a hypoplastic corpus callosum (arrow)

We report the case of a male child with severe NKH, currently alive at seven years of age - an unusually long survival for this form of the disease. He was born at term following an uneventful pregnancy. Fourteen hours after birth, he presented with feeding refusal, hypotonia, myoclonias, and hiccups. He developed a coma by the fifth day, requiring mechanical ventilation and transfer to the neonatal intensive care unit at a tertiary hospital. Brain MRI revealed a hypoplastic corpus callosum (Figure 1).

An electroencephalogram showed a burst-suppression pattern. Investigations revealed elevated glycine levels in plasma, urine, and CSF, with a markedly increased CSF/plasma glycine ratio, which confirmed a diagnosis of NKH. Detailed laboratory values are summarized in Table 1. The diagnosis was later genetically validated by identifying compound heterozygous mutations in the GLDC gene (c.1406G>A and c.2690G>C). A low-protein diet and glycine-lowering agents (such as sodium benzoate, levocarnithine, and dextromethorphan) were implemented. He was extubated on his 22nd day of life. The glycine plasmatic levels were normalized. He was transferred to his local hospital at 28 days of age and was later discharged home. Despite being treated with glycine-lowering medication and phenobarbital, his epilepsy remained uncontrolled, requiring frequent adjustments to his medication. His neurodevelopment was poor, with little social interaction, axial hypotonia and peripheral hypertonia, dystonic limb movements, and no acquisitions over time. He was put on nasogastric tube feeding due to swallowing difficulties. A gastrostomy was not performed due to the high anesthetic risk. He is permanently on noninvasive ventilation and uses a suction device and a cough aid.

**Table 1:** Patient’s laboratory parameters Reference ranges were obtained from the institutional clinical laboratory. CSF: Cerebrospinal fluid.

Laboratory parameters	Patient’s value	Reference range
Plasma glycine level	1,727 µM	223.8–514 µM
Urine glycine level	13,211.4 µM	283–1,097 µM
CSF glycine level	32.6 µM	3.7–7.6 µM
CSF/plasma glycine ratio	0.19	<0.04

This child has been under the care of an extended multidisciplinary team, including the pediatric palliative care team, metabolic diseases team, pediatric neurology, pediatric pulmonology, physical medicine and rehabilitation, dermatology, pediatric gastroenterology, ophthalmology, otolaryngology, orthopedics, general pediatrics, and social worker. Care was continuously coordinated between our tertiary hospital, his local hospital, and his general practitioner. He was started early on speech therapy, physiotherapy, and psychomotricity. Several pieces of equipment needed for his home care were arranged, such as a bath chair, car transport chair, and an articulated bed. His appointments were coordinated to take place on the same day to avoid unnecessary dislocation. The family also had direct lines of communication with the team, which enabled early identification and resolution of problems. Today, he has refractory epilepsy despite being medicated with three anticonvulsants; has no social interaction; does not walk, talk, or smile; and has peripheral spasticity. His parents eventually divorced, and his mother became his primary caregiver, which meant she had to leave her job. She is perfectly capable of providing the care her child needs.

## Discussion

NKH is a rare disease for which there is no cure, particularly for the severe form [1,2,3]. Although the medication reduces the severity of symptoms, it has no significant impact on developmental delay or refractory epilepsy [1].

According to Hübschmann et al., in severe NKH, symptoms appear earlier in life, with an onset before the age of three months and a CSF/plasma glycine ratio above 0.15 being specific for severe forms [4]. According to Swanson et al., patients with severe NKH require treatment with multiple anticonvulsants and experience therapy-resistant epilepsy; 71% of patients with severe NKH had brain malformations, and severe brain malformations such as corpus callosum agenesis occurred only in patients with a severe form. Only 12% of patients with severe NKH presented after the first week of life. Plasma and CSF glycine levels, as well as the CSF/plasma glycine ratio, were higher in patients with severe NKH than in those with attenuated NKH. The type of genetic mutation was also associated with disease severity [5].

Other studies have reported children with severe NKH alive at 6, 8, and 17 years of age, the latter bedridden [6-8]. There are cases of older individuals alive, with the oldest being 38 years of age, but the reports suggest that they have an attenuated form of NKH [9-11]. The majority of reported cases have resulted in neonatal or premature death [12-19].

The boy we describe appears to have a severe form of NKH: his symptoms began in the first week of life, he had very elevated plasma glycine levels and CSF/plasma glycine ratio, and he also had a poor developmental outcome and refractory epilepsy.

As mentioned above, there are a few reported cases of children with severe NKH who have survived for a long time [6-8]. Given the poor prognosis of severe NKH, this patient’s care was coordinated and centralized since an early stage, with the involvement of pediatric palliative care. We believe that this support for our patient and his family may have been responsible for prolonging his survival and quality of life of the children and the family. The whole team was always available to solve the problems that arose overtime, which led to early identification and resolution of problems, avoidance of hospitalizations whenever possible, and centralization of care. He was put on several therapies early on, which did not improve his neurodevelopment, as expected, but did improve his quality of life. His family was supported not only medically but also socially to integrate them into society and ensure that his needs and rights were met. The pediatric palliative care team was responsible for integrating and centralizing, and home visits were arranged to provide care at home. His parents were progressively empowered to care for him and were always compliant with the team, and they wanted to provide the best care for the child. At the same time, their wishes were always taken into account and integrated into the child’s care. With this coordinated palliative care, the patient remained stable over time despite the poor prognosis, with very few hospitalizations or clinical deteriorations. An advance care plan was written, shared with his family, and placed in his medical record in order to be available to health professionals in the event of clinical deterioration. We did not find any reports of support for other patients with the same condition.

## Conclusions

In conclusion, we present a boy with severe NKH with one of the longest survivals described. This disease has no effective treatment, and the prognosis is usually poor, with a lack of neurodevelopment progression, refractory epilepsy, and early death. The multidisciplinary palliative care provided to this patient and his family played an important role in prolonging his survival and improving his and his family’s quality of life.
